# A machine learning approach to predict self-protecting behaviors during the early wave of the COVID-19 pandemic

**DOI:** 10.1038/s41598-023-33033-1

**Published:** 2023-04-14

**Authors:** Alemayehu D. Taye, Liyousew G. Borga, Samuel Greiff, Claus Vögele, Conchita D’Ambrosio

**Affiliations:** 1 Department of Behavioral and Cognitive Sciences, University of Luxembourg, 4366 Esch-sur-Alzette, Luxembourg; 2 Luxembourg Institute of Health, 1445, Strassen, Luxembourg

**Keywords:** Psychology, Human behaviour, Risk factors

## Abstract

Using a unique harmonized real‐time data set from the COME-HERE longitudinal survey that covers five European countries (France, Germany, Italy, Spain, and Sweden) and applying a non-parametric machine learning model, this paper identifies the main individual and macro-level predictors of self-protecting behaviors against the coronavirus disease 2019 (COVID-19) during the first wave of the pandemic. Exploiting the interpretability of a Random Forest algorithm via Shapely values, we find that a higher regional incidence of COVID-19 triggers higher levels of self-protective behavior, as does a stricter government policy response. The level of individual knowledge about the pandemic, confidence in institutions, and population density also ranks high among the factors that predict self-protecting behaviors. We also identify a steep socioeconomic gradient with lower levels of self-protecting behaviors being associated with lower income and poor housing conditions. Among socio-demographic factors, gender, marital status, age, and region of residence are the main determinants of self-protective measures.

## Introduction

On 11 March 2020 the World Health Organization (WHO) declared coronavirus disease 2019 (COVID-19), the disease caused by severe acute respiratory syndrome coronavirus 2 (SARS-CoV-2), a global pandemic. During the first wave of the pandemic, without a vaccine or established therapeutic measures available, governments had to rely on behavioral interventions to slow the spread of the virus and reduce the number of infections. Authorities launched confinement policies—such as lockdowns, travel restrictions, and social distancing requirements—and preventive sanitary measures—such as mask wearing and frequent handwashing^1^. Even though it is apparent that human behavior largely influences the spread of the virus, it is unclear which factors are most strongly associated with protective behaviors. Identifying these factors is of paramount importance for devising effective policies to manage the current pandemic as well as to be better prepared for future ones^2,3^.

Using a machine learning approach, we examine how individual characteristics and government policy responses predict self-protecting behaviors during the earliest wave of the pandemic. We use a unique harmonized real‐time data set, the COME-HERE (COVID‐19, MEntal HEalth, Resilience, and Self‐regulation) longitudinal survey, collected by the University of Luxembourg in five European countries (France, Germany, Italy, Spain, and Sweden) covering a range of individual-level information such as sociodemographic variables, income and wealth, health and behavioral risk factors, awareness about the pandemic, and trust in major public institutions. In addition, we consult country level information describing the evolution of the pandemic itself, as well as the policy responses to COVID-19, from the Blavatnik School of Government of the University of Oxford COVID-19 government response tracker^4^.

Our work mainly relates to two strands of the literature. First, our study complements existing studies that try to understand self-protecting behaviors during the COVID-19 pandemic. Based on health behavior models, Aubert and Augeraud-Véron^5^ frame self-protection efforts as the outcome of a Nash equilibrium in which individuals choose the best response to their current economic, psychological, and epidemiological environment. Similarly, Brown and Ravallion^6^ posit that people trade-off the perceived benefit of social distancing against the cost, which includes costs in adjusting to the pandemic from a pre-pandemic level and the expected future cost of being infected.

In line with these model-based predictions, there is evidence suggesting that the most important determinants of self-protecting behaviors are socioeconomic factors. Papageorge and colleagues^7^ find that in the US, the costs of self-protective behaviors are unevenly distributed across socio-demographic groups such that economically disadvantaged individuals, who presume low risk of adverse health effects, are less likely to engage in protective behaviors. Ashraf^8^ shows a strong negative association between COVID-19 cases and socioeconomic conditions in a panel of 77 countries. Previous results also show that socioeconomic disparities in health behaviors create an imbalance between individual responses and socially optimal outcomes^9–11^.

Other factors influencing protective behavior that have been identified in the related literature include average neighborhood income^12^, internet access^13^, and political affiliation^14–16^. Other studies emphasize the role of perceived severity of COVID-19 and perceived susceptibility to it^17–19^, self-efficacy^20^, demographics^21^, and information source and credibility^22^.

The second strand of the literature that our paper relates to is the growing number of studies that apply a machine learning approach in social science investigations to detect fuzzy relations in complex data sets. Machine learning is a computational method that uses experience to improve performance on a given task. It follows a flexible algorithmic approach to learn the experience from the training data. The main focus of machine learning techniques is to perform prediction (regression) and classification, as well as clustering (grouping)^23^. For instance, recent studies in the social sciences have used machine learning methods to estimate how the effect of a particular intervention differs across characteristics of individuals^24–28^. With regards to the COVID-19 pandemic, a few studies employed machine learning methods to isolate and predict key indicators of compliance with social distancing rules^29^, fear and perceived health^30^, and psychological distress^31^.

Through the combination of these two research strands, our study makes several contributions to the existing literature. First, we use a combination of complex non-parametric machine learning model and state-of-the-art model explanation method to explain factors impacting the adoption of self-protecting behaviors during the COVID-19 pandemic. To the best of our knowledge this is the first attempt in the literature. Second, we demonstrate the advantages and relative gains of a tree-based algorithm over linear regression. Third, we train a highly predictive model with original data with a universe of items specifically constructed to measure behavioral change in response to COVID-19. This allows us to minimize the bias from measurement error, which is a common limitation in related studies that only focus on a few measures. Moreover, we use causal theory to justify the reason for including or omitting variables, and our data offer a large number of features. This enables us to minimize bias in the partial effects of features that may occur when an important variable is omitted in a causal model^32,33^. Fourth, our approach allows for the presence of interaction effects among key features. And finally, we identify key policy relevant individual and social predictors of self-protecting behaviors and document their heterogeneity by country.

## Data

Our study primarily relies on the COME-HERE survey as a data source. COME-HERE is a longitudinal survey conducted by a group of researchers at the University of Luxembourg to investigate the psychological and socioeconomic effects of the COVID-19 pandemic and related social distancing measures across Europe. The survey is administered by Qualtrics on nationally representative samples of individuals in France, Germany, Italy, Spain, and Sweden. This study was approved by the Ethics Review Panel of the University of Luxembourg (reference number: ERP 20-026-C COME-HERE). All research was carried out in accordance with relevant guidelines, and informed consent was obtained from all participants. Respondents were asked to complete an online questionnaire that took approximately 20 min. The survey collects information at the individual and household levels. The first wave of the survey took place in late April and early May 2020, and over 8000 people participated. The same pool of individuals was re-contacted nine additional times in June, August, and December 2020, in March, June, and October 2021, in February, June and December 2022 (see Vögele and colleagues^34^ for more information).

The present results are based on the first wave of the survey with over 8000 participants who answered a range of questions on their sociodemographic characteristics, socioeconomic variables, pre-existing health conditions and risk behaviors, mental health, resilience, social support, and trust in major institutions. This dataset is representative of the population in the countries in terms of age, gender and region of residence. In the next paragraphs, we outline in greater detail how we define the variables and features used in the prediction exercises.

Our outcome variable is a composite measure of self-protecting behaviors. The measure is constructed from a range of questions from the specially designed Coronavirus Behavior Scale ($CBS$). The $CBS$ is a 14‐item self-report measure of behavior change due to the Coronavirus pandemic. It contains two subscales, with nine items assessing reasonable behaviors (e.g., shaking hands less) and five items assessing unreasonable behaviors (e.g., buying more toilet paper than usual) that was particularly developed for the purpose of this and similar studies. Responses are given on a 5‐point Likert scale ranging from 1 strongly disagree to 5 strongly agree. We focus our analysis on the reasonable behavior subscale. This subscale is based on responses to the following questions: “Because of coronavirus, I am planning to or have already (1) cleaned and disinfected surfaces in my home more often, (2) bought medical masks, (3) stayed home when I feel ill, (4) started avoiding crowded spaces, (5) bought disinfectant, (6) started washing my hands more, and (7) shaken hands with people less”. We compute a composite index from the responses to the seven items of the reasonable $CBS$ subscale to define self-protecting behaviors of individual *i* $(CB{S}_{i}=\frac{1}{K}{\sum }_{k=1}^{K}ite{m}_{k})$.

We consider a host of sociodemographic, economic, and social contextual variables that predict self-protecting behaviors. These predictors include age, gender, marital status, education, employment status, household income and housing conditions, health status, behavioral risk factors (such as alcohol and cigarette consumption), confidence in institutions, and incidences of COVID-19 and responsive policy measures. In Supplementary Appendix A, we discuss in detail how each predictor variable is constructed and the necessary features pre-processing.

Table 1 presents the summary statistics of key characteristics of individuals in the pooled and country level samples. In the pooled sample, 8063 respondents were included, and the sample mainly consisted of working-age individuals (mean age of 47.5). Approximately 55.4% of respondents reported ties to the labor market (44% employed full-time and 11.5% part-time). Twenty percent of the sample are unemployed, and about 23.7% of the participants are retired. Nearly 58% are married and cohabiting, 8.7% are married but living apart, and 33.4% of the respondents are single. Close to 9% of the participants reported that they live in a house without any open-air access (such as garden, terrace, or balcony). Nearly 52% of respondents report at least one pre-existing health condition. In terms of household income, 21% of the respondents are in relative poverty. See Figure A.1 in Supplementary Appendix A for explorative correlation analysis between features.

**Table 1:** Descriptive statistics of selected variables.

	Pooled	France	Germany	Italy	Spain	Sweden
Gender (female = 1)	51.7	52.5	52.0	52.3	51.1	50.2
Age category						
[18, 20)	2.5	3.6	2.6	1.9	1.3	3.3
[20, 30)	16.3	14.9	13.7	23.2	14.2	15.3
[30, 40)	17.4	17.8	16.6	18.2	17.9	16.3
[40, 50)	17.9	17.8	16.2	18.2	21.4	15.5
[50, 60)	16.0	15.5	17.8	13.6	15.8	17.5
[60, 70)	19.3	20.5	22.4	16.5	20.8	14.9
70 and above	10.6	9.8	10.8	8.3	8.6	17.3
Family status						
Married & cohabiting	57.9	61.0	57.2	54.4	61.7	54.0
Married but living apart	8.7	3.9	9.6	13.5	8.9	7.6
Single	33.4	35.2	33.3	32.1	29.5	38.4
Employment status						
Full-time	43.9	47.7	44.8	40.1	46.9	38.7
Part-time	11.5	9.3	13.8	13.4	9.6	11.3
Unemployed	20.9	17.0	16.6	28.4	20.8	21.7
Retired	23.7	26.1	24.7	18.1	22.6	28.3
Education						
Low	7.8	6.7	4.0	9.3	10.1	9.4
Middle	37.5	38.6	28.3	51.6	22.7	49.9
Higher	55.0	54.7	67.7	39.1	67.2	40.7
Relative poverty	21.3	18.6	18.6	26.9	26.8	12.9
Housing feature						
Balcony	53.9	28.7	56.5	74.7	54.2	55.8
Park	4.4	4.9	3.9	4.4	3.7	5.6
Garden	47.1	61.4	54.0	45.8	24.3	50.9
Terrace	44.3	49.3	45.3	38.1	53.0	31.9
No home feature	8.8	11.4	7.9	3.7	13.3	7.2
No pre-existing illness	48.2	54.9	44.7	50.3	46.5	43.3
Confidence in						
Government	4.65 (1.82)	3.94 (1.79)	5.09 (1.49)	4.92 (1.74)	4.05 (2.00)	4.90 (1.88)
Health services	5.39 (1.41)	5.14 (1.43)	5.50 (1.29)	5.52 (1.36)	5.45 (1.43)	5.32 (1.57)
Essential services	5.66 (1.30)	5.35 (1.28)	5.78 (1.25)	5.81 (1.30)	5.88 (1.23)	5.44 (1.40)
Knowledge about COVID-19	5.24 (1.22)	4.90 (1.27)	5.25 (1.15)	5.58 (1.10)	5.38 (1.19)	5.04 (1.27)
CBS	3.86 (0.80)	4.01 (0.69)	3.54 (0.79)	4.19 (0.68)	4.04 (0.76)	3.40 (0.77)
N	8063	1706	1720	1710	1711	1216

This table presents summary statistics of respondents’ key characteristics and target variable in each sample used in the final prediction. The first column shows the summary statistics of selected variables of the pooled sample. The other columns list summary statistics of selected variables in each country. The numbers in the tables show the percentages. The figures followed by parenthesis are the mean and standard deviation of the variables, respectively. For the sake of presentation, the eight levels of education are recoded into three-level ISCED aggregation. CBS denotes the coronavirus behavioral scale.

## Method

Many features contribute to self-protecting behaviors against COVID-19. Our objective is to investigate this complex relationship with a high level of accuracy. We consider vectors of features capturing socioeconomic status (such as income and home features), pre-existing health conditions, health and behavioral risk factors, socio-demographic factors, severity of the disease and government policy responses, trust in major institutions, and knowledge about the pandemic.

Modelling this relationship with a parametric approach requires the researcher to make certain assumptions about the data-generating process, imposing structure on the data, which may eventually lead to functional misspecification. With supervised ML algorithm as used in this study, we do not need to assume any structure about the data-generating process but directly learn a function that maps the input features to the target variable. The learning process is controlled with hyperparameters that regulate some aspect of the learning algorithm. These hyperparameters are commonly optimized via a grid search over the hyperparameter space.

In this study, we build a Random Forests (RF) model and complement it with model-agnostic interpretative tools to ensure interpretability of our results along the lines of *explainable ML* (ExpML). See Fig. E.1 in Supplementary Appendix E for an overview of overall workflow. That is, we favor Random Forests (RF) over other ML algorithms because they are easily scalable to accommodate a large dataset with higher dimensionality without losing statistical efficiency. They are also shown to be state-of-the-art algorithms in tabular datasets with categorical variables^35^. Random forests can naturally capture complex interactions between features and their predictions are robust to outliers due to repeated sampling. RF is a collection of many *de-correlated* decision trees. Trees are grown on a bootstrapped sample with a random subset of feature vectors. In the next step, the final predictions are produced as a mean value of predictions from each tree (in a classification problem, the final predictions are yielded based on a majority vote). The steps in a typical RF algorithm are as follows: (i) Draw a bootstrap sample from the training data and randomly select *k* variables from *p* variables, where *k* <  < *p*. (ii) Select the best split among the *k* variables. The maximum number of *k* is a hyperparameter. (iii) Split the node into two daughter nodes. (iv) Repeat step *i* to *iii* until the terminal nodes have been reached— until no farther splits are possible. (v) Repeat the above steps for *T* number of times to grow a forest of *T* trees. In this study, all predictions are done with a popular library for the Python programming language called scikit-learn (version 0.22.2). Scikit-learn is an open-source project containing several state-of-the-art ML algorithms and their extensive documentation^36^.

We build a RF model consisting of 500 trees. To control over-fitting, we apply a grid search with fivefold cross-validation and identify the optimal hyperparameters. We optimize two important hyperparameters that will ensure randomness in RF: the maximum depths of trees (‘max depth’) and maximum number of features used in each split (‘max features’). The deeper the tree, the more splits it has, and it captures more information about the data. Similarly, by choosing a reduced number of features we can increase the stability of the tree and reduce variance and over-fitting.

The dataset is randomly divided into training (80%) and testing (20%) sets. For each combination of hyperparameters, we undertake the following three steps: (i) randomly partition the training set into five subsamples, (ii) train the models on the four subsamples and generate predictions for the fifth, and (iii) repeat this process five times so that each subsample is used only once to generate the prediction. The optimal hyperparameters are updated based on the average of these five prediction results. The tuning step ends when we find an optimal hyperparameter that produces minimum average prediction errors. For instance, the selected best RF model in the pooled dataset has a cross-validation test score of 0.7 Root Mean Squared Errors (RMSE) (The explored hyperparameters space, the cross-validation results, and the final hyperparameter combination used in the final model are available on request from the authors).

We assess the predictive accuracy of each Random Forests and a baseline OLS model because having a reliable estimate of predictive performance (e.g., a significant relative performance gain from using RF vis-a-vis the baseline model employing OLS) is an essential requirement for the socioeconomic interpretation of the results of this study. To quantify the extent to which the predicted value for a given respondent is close to the actual value of that individual, we use the most common metrics in regression settings: mean absolute errors (MAE) and root mean squared errors (RMSE). We use MAE to show how the model fares when prediction errors are linearly weighted. The best model will be the one that has a smaller value in both MAE and RMSE (ideally close to zero), meaning that it produces predictions that are close to the true responses. In our data, both metrics produce similar conclusions.

We find that the RF model outperforms OLS in all prediction tasks in both the pooled and per-country datasets (see Table D.1 in the Supplementary Appendix for detailed results). The superior prediction performance of RF shows that we could capture signals relevant to self-protecting behavior, which the linear model failed to capture (as measured by the percentage reduction of the prediction error).

Finally, we use the visualization tool SHapley Additive exPlanations (SHAP) proposed by Lundberg and Lee^37^ to explain the contribution of each feature to the prediction of self-protecting behaviors using Shapley values. SHAP is based on a solution concept in a cooperative game setup that aims to ‘fairly’ allocate the gains among players as suggested in the seminal work of^38^. SHAP has the advantage of consistency and provides both local and global interpretability^36,39,40^.

## Results

### Identifying the top 30 predictors

**Figure 1:**
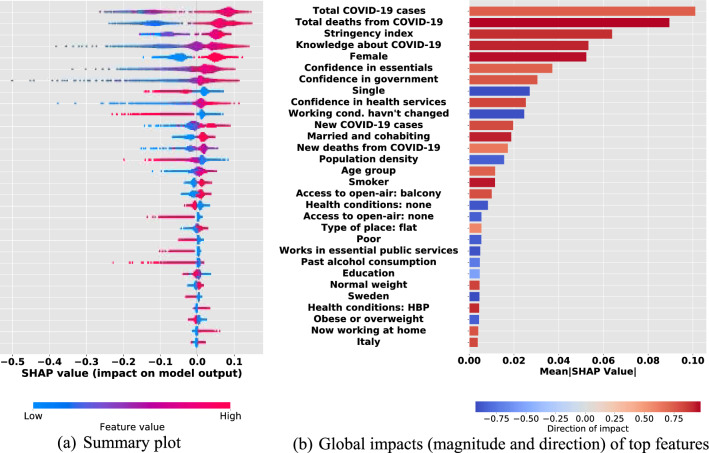
Top 30 predictors of self-protecting behaviors. *Notes*: Panel (**a**) is the SHAP summary plot for the Random Forests trained on the pooled data set of five European countries to predict self-protecting behaviors responses against COVID-19. The plot displays the top 30 features on prediction (the top on the y-axis is the most important) and the distribution of the impacts of each predictor on the model prediction, which includes a set of distributions where each dot corresponds to an individual. When multiple dots arrive at the same coordinate in the plot, they pile up to show density. The colors correspond to the feature values: red for larger values and blue for smaller ones. A negative SHAP value (extending to the left) shows reduced self-protecting behavior, while a positive (extending to the right) shows an increased self-protecting behavior. Panel (**b**) displays threefold information: (i) the direction of association captured by the correlation between the feature and SHAP values (red for positive and blue for negative); (ii) strength of the direction of association shown by the darkness of each color gradient; (iii) the magnitude of feature’s marginal impact measured as the average of absolute SHAP values.

We identify the top 30 features in predicting self-protecting behaviors. Figure 1 panel (a) presents a SHAP summary plot that succinctly displays the importance of the 30 features identified, the magnitude of their impact (i.e., the effect size), and the direction of a specific feature’s association with self-protecting behaviors (see Fig. B.1 in Supplementary Appendix B, for detailed results showing all features used in the prediction exercise). On the y-axis, the top 30 predictors of self-protecting behaviors are positioned in descending order of average contribution to the prediction (i.e., the global contribution measured in mean absolute SHAP value). On the x-axis the SHAP values for each observation are presented—negative SHAP values are interpreted as reduced self-protecting behavior, while positive SHAP values are interpreted as increased level of self-protecting behaviors.

Each dot represents an individual respondent; hence, the number of dots against each feature reflects the sample size of the training set. The dot’s position along the x-axis is the feature’s impact on the model’s prediction for that respondent. When multiple dots arrive at the same coordinate in the plot, they pile up to show the density of effect sizes. The long-left tails in the summary plot indicate that the predictors are highly predictive for some respondents but not others, i.e., predictors with minor global importance can still be very important for specific respondents.

In Fig. 1 panel (b), we summarize our key findings for an easier global explanation of the impact of the features on the model and their association with self-protecting behaviors. The horizontal length of each bar shows the magnitude of impact on the model. The correlations of each feature with self-protecting behaviors are shown by the color code of each bar. We can see that features such as total number of deaths from COVID-19, stringency index (see Supplementary Appendix A for the variable definition), knowledge about COVID-19, being female, being married and co-habiting, having a pre-existing high blood pressure (HBP) and being smoker (including those who currently smoke or ex-smokers) have strong positive associations (correlation coefficient ≥ 0.9) with self-protecting behaviors, whereas being single, not experiencing COVID-19 induced work-related changes, not having pre-existing health conditions, not smoking, residing in Sweden, and working in essential public services all show strong negative associations (correlation coefficient ≤  − 0.9) with self-protecting behaviors. We also perform a robustness check of feature ranking using an alternative method to SHAP, feature importance (see Fig. C.1 in Supplementary Appendix C for feature ordering using permutation feature importance). Note that there is a big difference between these two alternative methods: permutation feature importance is based on the decrease in model performance whereas SHAP is based on the magnitude of feature attributions.

In the next subsections, we examine how each of the top 30 features contributes to the model’s output. Since self-protecting behaviors are likely to depend on locally relevant perceptions of threats, susceptibility, and benefits in engaging in protective behavior, we structure our results according to factors related to epidemiological, socioeconomic, and specific health conditions. As most of the predictors of self-protecting behaviors are categorical, we create box plots to show the difference in the average marginal contribution of each category in a more robust way. The y-axis of the box plots shows the SHAP value of the variable, and on the x-axis are the values that the variable takes. We then systematically investigate interactions between features which have some policy relevance. In the end, we report heterogeneity analysis by country.

### Epidemiological factors

Epidemiological models of the spread of infection diseases postulate that people start reacting against contracting a disease with self-protective measures whenever they are informed about the disease and when the burden of the disease is at a recognizable stage^41^. The variables typically used in epidemiological models include population density, stringency of lockdown measures, number of new infections and fatalities, confidence in institutions, and knowledge about the specific disease, COVID-19 in this case. Our results confirm that these indicators are all important predictors of self-protecting behavior.

**Figure 2:**
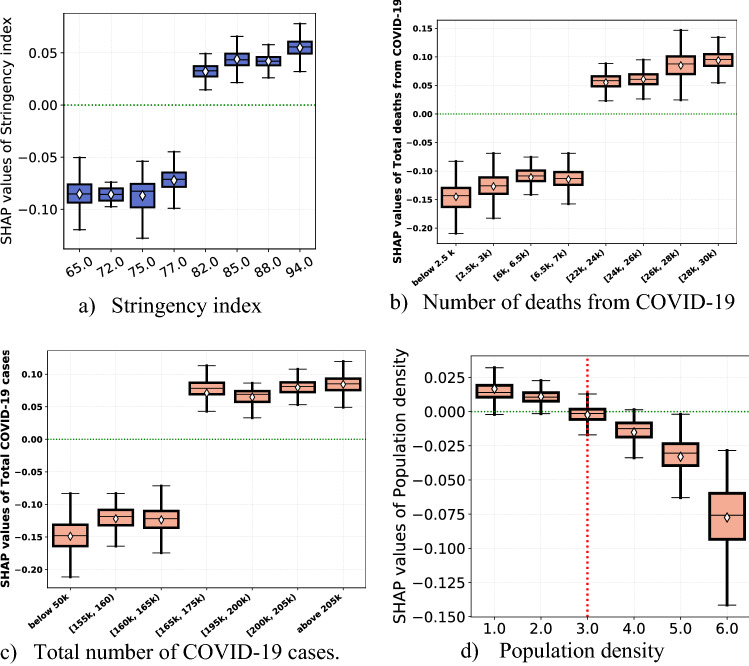
Partial effects of stringency policy response and local infection rate. *Notes*: This figure displays SHAP dependence boxplots of the stringency index. The diamond symbol in the boxes denotes the average of the SHAP value distribution per each value of the stringency index during the first wave of the pandemic. In panel (**d**), the labels in the x-axis correspond to the number of people in the respondent’s residential area, (1) “isolated dwelling”, (2) “less than 2000”, (3) “between 10,000 and 2000”, (4) “between 50,000 and 10,000”, (5) “between 100,000 and 50,000”, and (6) “more than 100,000”. The values in the x-axis of panels (**b**) and (**c**) are the number of deaths and cases summarized in a few bins.

Figure 2 depicts the effects of local case and death rates and the national policy responses on self-protecting behaviors. Our results show that total number of cases and deaths linked to COVID-19, and the OxCGRT stringency index are ranked first, second, and third most important predictors of self-protecting behaviors respectively. A striking positive non-linear pattern exists between the stringency index and self-protecting behaviors. From panel (a) of Fig. 2, we see that a stringency index of 80 (with the index ranging between 0 and 100, 100 = most stringent response) is an important threshold over which any increase in strictness of policy response triggers a level of self-protecting behaviors higher than the mean prediction (positive SHAP values).

Panels (b, c) of Fig. 2 contain the partial effects of infection rate on individuals’ behavior. Both measures of infection rate show a step like association with self-protecting behaviors. Behavioral responses remain quite unresponsive with respect to national infection rates for a while and jump higher following the total number of deaths from COVID-19 spiking up to above 20,000 from somewhere below 7000 deaths. From panel (d), we observe a somewhat non-linear negative association between the degree of population density and the level of self-protecting behaviors. Overall, living in a densely populated area/city/town/village is associated with lower self-protecting behaviors.

**Figure 3:**
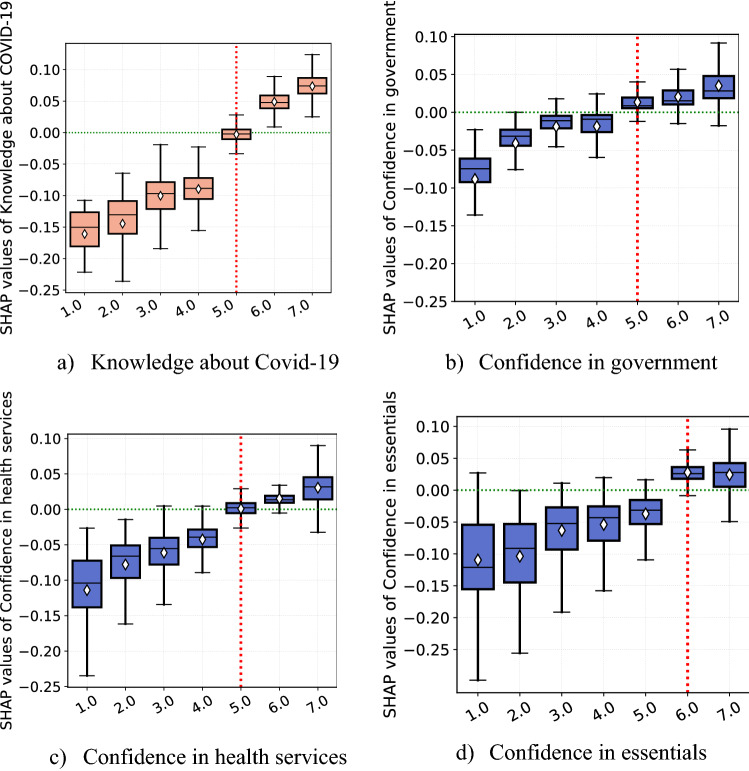
Partial effect of confidence in institutions and level of knowledge about COVID-19. *Notes*: Each panel (**a**)–(**d**) displays SHAP dependence boxplots of features related to trust in institutions and knowledge about COVID-19. The diamond symbol in the boxes denotes the average of SHAP value distribution per each category.

Confidence in the essentials, government, and health services are the top 6^th^, 7^th^ and 9^th^ most important features in our model, respectively. We find a consistent positive association between confidence in the essentials, government, health services, and the SHAP of each feature with correlation coefficients of 0.74, 0.77, and 0.84, respectively (Fig. 3 panels (b)–(d)). We also identify an important level of confidence threshold (shown by the red dotted vertical line in the panels) over which the three features about confidence in major institutions are associated with positive SHAP values. We find higher threshold for confidence in essentials (degree of confidence = 6), compared to the other two features (degree of confidence = 5).

Panel (a) of Fig. 3 reveals a strong positive relationship between self-assessed level of respondent’s knowledge about COVID-19 and level of self-protecting behaviors. It is the 4th most important predictor of self-protecting behaviors. We discover an important threshold (level of knowledge = 5) over which the level of knowledge is associated with positive SHAP values.

Interestingly, all features relating to confidence in authorities and knowledge about COVID-19 have long left tails shown in the summary plot, suggesting that the lower degree of trust and awareness about the pandemic is exceptionally detrimental to self-protecting behaviors as compared to a higher level of confidence is in enhancing protective behavior.

### Socioeconomic factors

**Figure 4:**
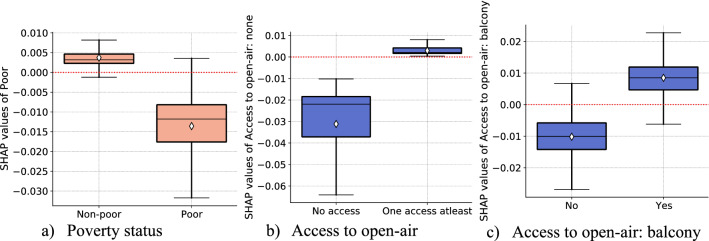
Partial effects of income and housing features. *Notes*: Each panel (**a**)–(**c**) display SHAP dependence boxplots of income and housing features. The diamond symbol in the boxes denotes the average of SHAP value distribution per each category.

Our results identify a strong socioeconomic gradient in self-protecting behaviors. Panels (a)–(c) of Fig. 4 present partial effects of key socioeconomic features on self-protecting behaviors. Results depicted in panel (a) show a strong negative association between the poverty status and the level of self-protecting behaviors. On average, the non-poor have a self-protecting behaviors level that is higher than the mean prediction of the model $\left(E(\widehat{f}\left(x\right))=3.866\right)$ while the poor exhibit a level of self-protecting behaviors smaller than the average prediction of the model. Hence, the economically better-off individuals (those non in poverty, that is, individuals with an equivalent household income above 60% of the country’s median) tend to report a higher level of engagement in protective behaviors against COVID-19. This suggests that adherence to some sanitary recommendations (e.g., physical distancing) is a costly option to relatively poor households. Hence, authorities should devise a buffering mechanism that could enable the financially vulnerable individuals to cope with the economic burden of pandemic health behaviors.

We also find that access to open-air at home are among the strong predictor of self-protecting behaviors and they positively impact respondents’ protective behaviors. We assume that better home features are indicators of higher wealth. RF prediction shows that respondents that live in homes without access to open-air have, on average, self-protecting behaviors that are well below the mean prediction. Having access to open-air with at least one home feature guarantees the level of self-protecting behaviors that is above the mean prediction. Of the different home features considered in our model, having a balcony appears the most important in terms of relative contribution to the model, whereas access to garden, terrace, and park do not feature in the top 30. The reason that balcony is among the main factor determining people's behaviors is that during COVID-19, it is through their balconies that people enjoy at least some sense of being outdoors while confined at home. Its functionality as a result has been diversified to be as a work corner, a place to exercise, a place of contemplation, and fresh air without the anxiety of breaking the lockdown rules.

Housing features, such as access to open-air and living in accommodation that are not overcrowded, enable individuals to comply with protective measures better. In addition, lack of access to outdoor spaces affects dwellers' mental health during the pandemic influencing decisions to engage in protective behaviors^42^. Hence, precautions may seem more relevant in dense localities and poor living arrangements. Local authorities should prioritize devising temporary open spaces accessible to residents during pandemics. The long-run housing policy should also prioritize open-air access as an integral part of housing design.

### Specific health conditions

**Figure 5:**
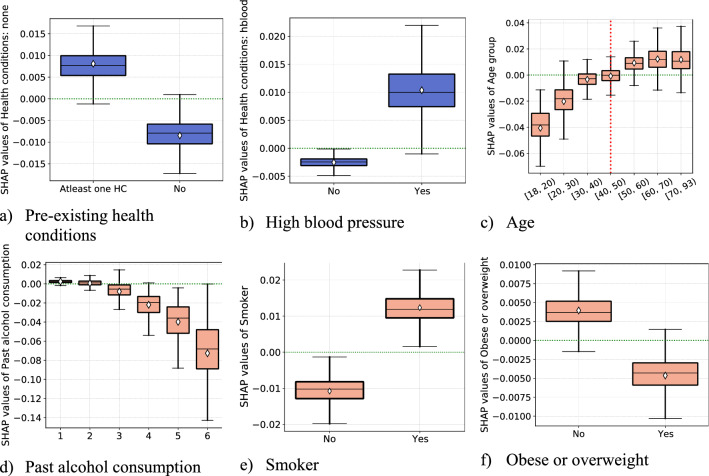
Partial effects of pre-existing health conditions and behavioral risk factors. *Notes*: Each panel (**a**)–(**f**) display SHAP dependence boxplots of pre-existing health conditions and behavioral risk factors. The diamond symbol in the boxes denotes the average of SHAP value distribution per each category. In panel (**d**), the labels in the x-axis correspond to the number alcohol consumption in number of glasses in an average week: (1) “ < 5”, (2) “[5, 10)”, (3) “[10, 15)”, (4) “[15, 20)”, (5) “[20,25)”, (6) “ ≥ 25”.

The role of specific health conditions has been emphasized regarding the severity of COVID-19 once infected^6^. We also find that having at least one pre-existing health condition is strongly associated with higher self-protecting behavior, as shown in panel (a) of Fig. 5. Moreover, we assess how each pre-existing condition fares in terms of contributing to self-protecting behaviors prediction. As evident from panel (b), respondents with the pre-existing condition of high blood pressure tend to engage in a higher level of self-protecting behaviors than the others.

Panels (c)–(e) of Fig. 5 display the impact of health and behavioral risk factors (described in “Data”) on the overall model prediction. We find evidence that past alcohol consumption and obesity are negatively related to self-protecting behaviors. There is strong evidence that alcohol consumption is negatively associated with self-protecting behaviors. Obesity is associated with increased risk of severe illness from COVID-19^43,44^. At the same time, obesity is strongly related to low socioeconomic status^45^, that may explain the negative relationship between obesity and self-protecting behaviors.

Although smoking is an adverse health behavior, interestingly, our results show that smokers (including those who currently smoke or ex-smokers) tend to manifest a higher level of self-protecting behaviors than non-smokers. Hence, this increased risk of COVID-19 for smokers might motivate them to exhibit higher compliance. Consistent with Dai and colleagues^46^, who report that smokers tend to suffer severe outcome of COVID-19, whereas alcohol consumption was not linked to severe complication from the virus, we find that alcohol consumption deos not motivate greater self protection. Similarly, other study argue that smokers tend to have more severe symptoms of COVID-19 compared to non-smokers^47^.

At the beginning of the pandemic older people have been found to be disproportionately more likely to have severe symptoms from COVID-19 leading to hospitalization and death. We find that the nature of the relationship between age and self-protecting behaviors is somewhat non-linear, i.e., for age groups older than 50 years of age, we do not find a significant difference in the average level of self-protecting behaviors. Moreover, 40 years of age appears to be an essential threshold over which the number of years is associated with positive SHAP values.

### Sociodemographic factors

**Figure 6:**
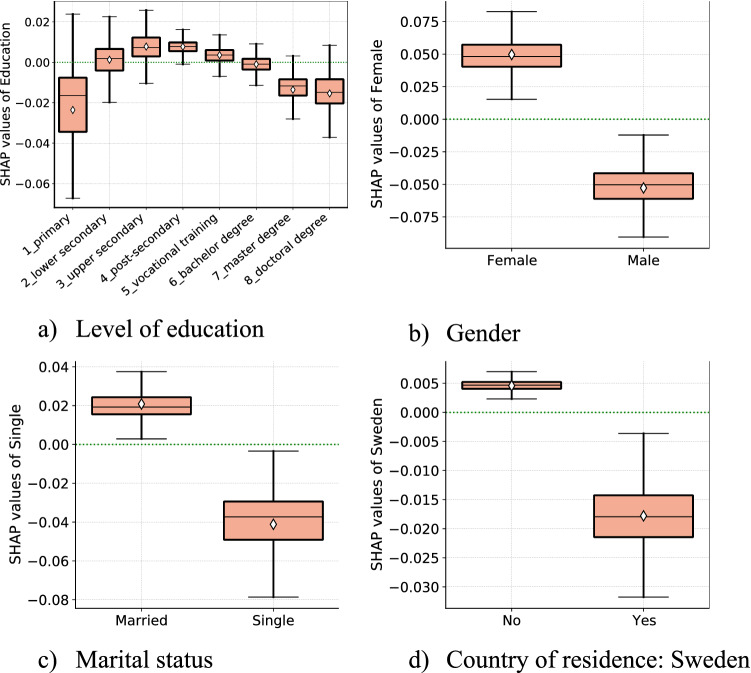
Partial effects of socio-demographic factors. *Notes*: Each panel (**a**)–(**d**) display SHAP dependence boxplots of socio-demographic features. The diamond symbol in the boxes denotes the average of SHAP value distribution per each category.

The next set of features we consider are the main socio-demographic indicators, such as gender, family status, country of residence and level of education of respondents. In panel (a) of Fig. 6, we examine the partial effect of individuals’ level of education and their corresponding self-protecting behaviors. We do not find a monotonically increasing protective behavior with educational attainment. Our results show a non-linear association between education and self-protecting behaviors: the lowest level of self-protection is associated with the lowest education group. It peaked with the middle education group and then slightly decreased for the highest education group. The increasing part (in the partial effect of education) re-affirms the strong association between socioeconomic status and self-protective behaviors. The slight drop in self-protection from middle to the highest education level could be explained by differential exposure to the pandemic for the high socioeconomic group. One such important difference is remote work. For instance, in our data, 36% of individuals with higher education levels responded that they were working at home during the first wave of the pandemic. This remote working arrangement is disproportionately higher for the more educated groups than the middle (16.5%) and lower (6%) education groups. Thus, it is plausible to argue that individuals with higher educational attainments have a low risk of getting infected with COVID-19 in the first place than those with low and middle educational attainment^48^.

Our model identifies gender as the 5th top predictor of self-protecting behaviors. As evident from panel (b) of Fig. 6, there are substantial gender differences in health-protective behaviors. Females tend to exhibit a higher level of self-protective behavior. Gender differences in fear and risk perception can potentially explain this pattern^49^. The intra-household bargaining theory can also explain the residual effect—where women engage more in household caretaking roles while men continue to work in person^50–53^.

From panel (c) of Fig. 6, we see the partial effects of family status of individuals in determining the level of self-protecting behaviors in our model. Single respondents (ranked 8th) react less to the pandemic. This study also isolates a positive, partial impact of being married and cohabiting vs. married but living apart. Past work documented that loneliness is linked with lower engagement in COVID-19 protective behaviors, suggesting that individuals who live alone could find it more challenging to restrain themselves from meeting others as they lack companionship at home^54^.

In panel (d) of Fig. 6, we present the partial effect of country of residence on the level of self-protecting behaviors. The results show that compared to respondents from Italy, Spain, France, and Germany, respondents from Sweden tend to exhibit lower level of self-protecting behaviors during the first wave of the pandemic. In fact, Sweden was the only country never in lockdown.

### Measuring interaction effects

In the preceding sections we highlighted the top-ranking features that are predictive of self-protecting behaviors. In some cases, it is plausible to assume that the relationship between the target variable and a feature depends on the value of another feature. Such contextual dependence between features that jointly impact predictions are known in the ML literature as feature interactions. Our RF model automatically accounts for these interactions, and we now systematically report interaction effects that have some policy relevance.

**Figure 7:**
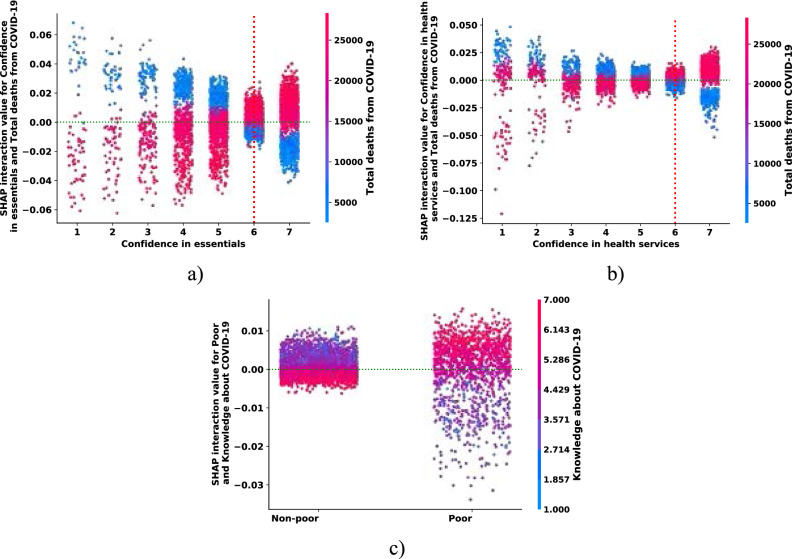
Feature interaction effects. *Notes*: Each panel (**a**)–(**c**) displays SHAP feature dependence plots of the RF model with the largest interaction effect. Artificial jitter (0.5) was added along the x-axis to better show the overlapping distribution of the points.

The interaction between confidence in essentials with the number of deaths from the COVID-19 shows that the relationship between self-protecting behaviors and confidence in essentials is alerted by the national incidence rate of the pandemic, observed in Fig. 7 panel (a). Those individuals who are highly confident (level of confidence on the Likert scale six and above) that essential services will be maintained during the pandemic exhibit a higher level of self-protecting behaviors proportional to the number of deaths. On the other hand, those respondents with lower confidence in essentials respond asymmetrically to the pandemic's national incidence rate. We find a similar impact on confidence in health services as seen in Fig. 7 panel (b).

Next, we investigate the interaction effect between knowledge about COVID-19 and the relative poverty status of individuals: in Fig. 7 panel (c), we see a more pronounced positive effect of knowledge about the pandemic and self-protective behaviors among the poor group than the non-poor, meaning that among the poor those who show a higher level of self-protection are the ones who have better awareness about the pandemic. However, in the non-poor groups, the interaction effect is slim, and self-protection behaviors are not necessarily tied to the level of knowledge. This result is in line with the partial effect of education examined above. It is safe to assume that a high level of education and better knowledge about COVID-19 are highly correlated.

These interaction effects imply that enhancing an individual’s trust in institutions could enable people to show behavioral responses with respect to the national incidence rate. Moreover, awareness creation about the pandemic that targets the low socioeconomic group could result in a better behavioral response.

### Heterogeneity by country

Detailed result of feature importance and direction of association in each of the five countries separately is presented in Figs. B.2 and B.3 in Supplementary Appendix B. Confidence in public institutions, self-assessed knowledge about COVID-19, gender, age, and marital status are the feature among the top ranked predictors of self-protecting behaviors in each country. Consistent with the results from the pooled sample, COVID-19 infection and death counts also appear among the top 30 predictors in all countries.

## Discussion

In response to the COVID-19 pandemic, several national governments have applied lockdown restrictions and requested their citizens to undertake some behavioral changes to reduce the infection rate. Even though it is apparent that human behavior largely influences the spread of illness, it is unclear what factors most strongly correlate with protective behavior. We examine the most relevant predictors of individual self-protective behavioral responses during the first wave of the COVID-19 pandemic employing a data-driven approach of ExpML on real-time survey data from five European countries.

Employing state-of-the-art machine learning approaches, we show that COVID-19 disease progression and the responses that governments give through policies induce an increase in self-protecting behaviors provided that both the number of COVID-19 infection/death rate and the strictness of policies are sufficiently high. The government policy setting and the local infection rates play an important role in individuals' responses to the health crisis. These factors guide how individuals adopt the protective guidelines. Higher local infection rates increase people's perceived risk of getting infected, inducing responses to protective behaviors. This was further reflected in the macro features relating to the country of residence. Compared to respondents in Sweden, respondents from Italy, Spain, France, and Germany tended to exhibit a higher level of protecting behavior during the first wave of the pandemic. This can be explained by the stringent lockdown measures in these countries in April and early May 2020. The Swedish government promoted individual responsibility rather than mandatory restrictions during this period.

Our findings shed light on some social and economic features that policymakers could modify to bridge the socioeconomic gap that we identified in self-protecting behaviors. Consistent with economic models that predict suboptimal individual behavior in the presence of strong externalities where the costs of protective behavior are unevenly distributed across socio-demographic groups, we find that people with lower socioeconomic status are less likely to adhere to protective behaviors. First, we show that increased knowledge about COVID-19 and trust in government, health care, and essential services is crucial to adopting protective behavior. Second, we find that a lower level of self-protecting behaviors is associated with lower income. Third, we observe that suitable living arrangements and localities are positively related to health-protective behaviors.

### Limitations

It is important to keep in mind some limitations when interpreting the findings of this study. First of all, many complex ML algorithms might, on the one hand, be highly predictive but might, on the other hand, lack intrinsic model interpretability. To circumvent this issue, we employed ExpML that has the advantage of providing an opportunity to improve prediction accuracy over standard OLS using a ‘black-box’ supervised ML algorithm while still revealing interesting insights into a complex outcome.

More specifically, we built a RF model and complement it with model-agnostic interpretative tools. Related to this first limitation, interpretability and relative contribution of each predictor are influenced by the cross-sectional nature of the data preventing any causal conclusions on the basis on this data set. It is also important to keep in mind that we analyze the early wave of the pandemic (i.e., Spring 2020) and without a vaccine available. Both the fact that more knowledge on the pandemic emerged over time and that a vaccine became available might have had an impact on the factors that drive self-protecting behaviors. We also only report a limited number of interactions between features out of all possible binary combination among them and limited our analyses to those interactions that we assumed to have the highest levels of policy relevance. Finally, overfitting poses a problem in the current study. To this end, we applied a five-fold cross-validation and identified an optimal set of hyperparameters (i.e., maximum depths of trees and maximum number of features per split) to reduce the potential impact of overfitting.

## Conclusions

Key findings of this paper shed light on essential policy variables that help to slow the current pandemic and guide the design of future policies with important implications for researchers and policy makers. Effective communication by authorities that restores trust, providing basic support to the financially disadvantaged, increasing access to open air for those residing in houses that lack basic outdoor features are some of the policy levers that government could pull to incentivize changes in protective behavior.

## Supplementary Information

Supplementary Information.

## Data Availability

The COME-HERE data contain sensitive individual information and cannot be made publicly available (Ethic Research Panel of the University of Luxembourg, decision number ERP 20-026 C/A COME-HERE). Data access to the University of Luxembourg server can be obtained on request for non-commercial research purposes by contacting the corresponding author. The code used in the analysis is available at 10.5281/zenodo.7788267.
