# Supplementary material for: A machine learning approach to predict self-protecting behaviors during the early wave of the COVID-19 pandemic
Source: Sci Rep. 2023 Apr 14;13:6121. doi: 10.1038/s41598-023-33033-1 (PMC10103659; doi:10.1038/s41598-023-33033-1)
Supplement: Supplementary file 1 — Supplementary Information. [file 41598_2023_33033_MOESM1_ESM.docx]

**Supplementary Information for:**

**A machine learning approach to predict self-protecting behaviors during the early wave of the COVID-19 pandemic**

Alemayehu D. Taye^1^, Liyousew G. Borga^1,2^, Claus Vögele^1, *^, Samuel Greiff^1^, Conchita D’Ambrosio^1^

^1^Department of Behavioral and Cognitive Sciences, University of Luxembourg, 4366, Esch-sur-Alzette, Luxembourg

^2^ Luxembourg Institute of Health

^*^Corresponding author. Email: Claus.Voegele@uni.lu

**A. Predictors of self-protecting behaviors**

**Sociodemographic factors**: We consider gender, age, marital status, education, employment status, and residence. Age is recoded into seven-level ordinal categories, such as individuals below 20 years of age, five categories between 20 to 70 years of age, each spanning a range of 10 years, and individuals older than 70. Marital status is recoded into three level non-ordinal variables to allow us to understand the effect of cohabitation: single (includes individuals who never married, divorced, or widowed), married and cohabiting, and married but living apart. The highest level of educational attainment was measured with eight levels (primary, lower secondary, upper secondary, post-secondary, vocational training, bachelor’s degree, master’s degree, and doctoral degree).

The respondents’ ties to the labor market were measured with four binary variables, employed full-time, employed part-time, retired, and unemployed. Furthermore, respondents were asked if they have been working in any ‘key sectors’ defined by governments. We recoded these measures into nine binary features: essential public services; energy and oil; health; food sector; water distribution; waste removal; finance and insurance; and transport. Moreover, respondents were also asked to report changes in employment status, work arrangements, and job losses since the implementation of social distancing. We measure the population density of the respondents’ living area with a six-level ordinal variable according to the inhabitants in the living area: 1 “Isolated dwelling”, 2 “less than 2,000”, 3 “between 2,000 and 10,000”, 4 “between 10,000 and 50,000”, 5 “between 50,000 and 100,000”, and 6 “more than 100,000”.

**Income and housing**: Respondents were asked to record their net monthly disposable household income in January 2020 with the following income bands (all in Euros): 0-1250; 1250-2000; 2000-4000; 4000-6000; 6000-8000; 8000-12500; and >12500. A “prefer not to say” option was available as an alternative answer. Following Clark et al. (2021) we take the mid-point of each band and adjusted it for purchasing power using 2019 Euros for household final consumption expenditures as the reference. We assign 15000 Euros to the open-ended top income band. To account for economies of scale across members of the same household, each income figure is equivalized using the square root of family size, and the resulting value is attributed to each household member. We then compute the relative poverty status of individuals based on the poverty line defined as 60% of the median of the equivalent income distribution of the country.

We capture features relating to dwelling characteristics, such as housing type (with six categories), and the existence of an outdoor space in the housing (housing features, e.g., balcony, terrace, garden). We also compute a binary variable to capture the household-level overcrowding (1 = if the number of required rooms is bigger than the number of current rooms in the household), following the Eurostat definition (see OECD, 2021, https://data.oecd.org/inequality/housing-overcrowding.htm.)

**Health conditions**: Participants were asked to report if they had pre-existing chronic diseases such as pollen allergy, diabetes, high blood pressure, heart disease, lung disease, or asthma, cancer, and disability. Respondents were also asked if they had ever been clinically diagnosed with any mental disorders in the past and during the last two weeks.

**Health and behavioral risk factors**: We consider indicators for smoking, drinking, and BMI measures. Smoking was assessed by a binary variable, which takes value one if the respondents are currently smoking or were ex-smokers, and value zero if the respondents are non-smokers. Alcohol consumption is measured in the number of glasses in an average week and during the last week. BMI was calculated based on the weight and height of the respondents and categorized into three categories: “overweight or obese” if BMI ≥ 25; “normal” if 18.5 ≤ BMI < 25; and “underweight” if BMI < 18.5. In addition, we use a dummy variable measuring if respondents take any medication in a higher dose or frequency than prescribed.

**Confidence in institutions and knowledge about COVID-19**: Respondents were asked to rate on a 7‐point Likert scale (ranging from 1 = “not at all confident” to 7 = “full confidence”) their degree of confidence that 1) the government can handle COVID‐19 well, 2) the health services can cope during COVID‐19, and 3) essentials will be maintained during COVID‐19. Individuals were also asked to rate their level of knowledge about COVID‐19 on a 7‐point Likert scale with higher scores indicating better knowledge.

**Incidences of COVID-19 and policy responses**: We consult the Oxford COVID-19 Government Response Tracker (OxGRT) to access country-level information on the government policy response to COVID-19 and variables measuring the incidence and evolution of the pandemic (Hale et al., 2021). We use the number of total and new COVID-19 cases and deaths to measure the evolution of the pandemic. The stringency index is a composite measure based on nine response indicators that gauge the strictness of the confinement measures of governments, such as: “school closures”; “workplace closures”; “cancellation of public events”; “restrictions on public gatherings”; “closures of public transport”; “stay-at-home requirements”; “public information campaigns”; “restrictions on internal movements”; and “international travel controls”. We merge this information with the COME-HERE data by date of interview to maintain variation at the individual level.

Table A.1: Demographic and socioeconomic characteristics of the sample compared to national statistics

|  | France* | Germany | Italy | Spain | Sweden |
| --- | --- | --- | --- | --- | --- |
| Median age | ﻿47.2 [42] | 49.0 [46] | 45 [47] | 48 [44.3] | 49 [40.5] |
| Gender (percentage of female) | 52.5 [52] | 52.0 [51] | 52.3 [51.3] | 51.1 [51] | 50.2 [50] |
| Relative poverty rate | 18.6 [14.2] | 18.6 [16.1] | 26.9 [20.0] | 26.8 [21.0] | 12.9 [16.1] |
| Regional distribution: |  |  |  |  |  |
| Parisian Region (Île de France) | 18.2 [18.3] |  |  |  |  |
| Paris Basin | 16.2 |  |  |  |  |
| West | ﻿14.1 |  |  |  |  |
| Mediterranean | ﻿13.1 |  |  |  |  |
| East Central | ﻿12.1 [12.0] |  |  |  |  |
| South-West | ﻿11.1 |  |  |  |  |
| East | ﻿9.1 |  |  |  |  |
| North | 6.0 |  |  |  |  |
| Baden-Württemberg |  | 12.2 [13.35] |  |  |  |
| Bayern |  | 15.0 [15.78] |  |  |  |
| Berlin |  | 4.0 [4.41] |  |  |  |
| Brandenburg |  | 3.02 [3.03] |  |  |  |
| Bremen |  | 1.0 [0.82] |  |  |  |
| Hamburg |  | 3.02 [2.22] |  |  |  |
| Hessen |  | 8.0 [7.56] |  |  |  |
| Mecklenburg-Vorpommern |  | 2.0 [1.93] |  |  |  |
| Niedersachsen |  | 9.6 [9.61] |  |  |  |
| Nordrhein-Westfalen |  | 21.7 [21.58] |  |  |  |
| Rheinland-Pfalz |  | 5.0 [4.92] |  |  |  |
| Saarland |  | 1.3 [1.19] |  |  |  |
| Sachsen |  | 5.0 [4.90] |  |  |  |
| Sachsen-Anhalt |  | 2.7 [2.64] |  |  |  |
| Schleswig-Holstein |  | 3.5 [3.49] |  |  |  |
| Thüringen |  | 2.9 [2.57] |  |  |  |
| Northwest |  |  | 27.3 [27] |  |  |
| Northeast |  |  | 18.4 [19] |  |  |
| Centre |  |  | 20.1 [20] |  |  |
| South |  |  | 23.2 [23] |  |  |
| Islands |  |  | 11.1 [11] |  |  |
| Northwest |  |  |  | 10.1 [9.1] |  |
| Northeast |  |  |  | 9.9 [9.5] |  |
| Community of Madrid |  |  |  | 14.1 [14.3] |  |
| Centre |  |  |  | 13.0 [11.6] |  |
| East |  |  |  | 29.2 [29.4] |  |
| South |  |  |  | 20.4 [21.4] |  |
| Canary Islands |  |  |  | 3.4 [4.7] |  |
| East Sweden |  |  |  |  | 39.0 [40] |
| Southern Sweden |  |  |  |  | ﻿44.1 [43] |
| North Sweden |  |  |  |  | 17.1 [17] |

*Notes*: This table presents comparison of our sample to [overall national statistics obtained from Eurostat]. Relative poverty rate is based on the poverty line defined as 60% of the median of the equivalent income distribution of 18 years of age and above in the country.

* The number of regions (regional aggregation) in the Eurostat database is not exactly aligned with our sample for France.

Figure A.1: Correlations between features

*Notes:* This figure presents pre-ML analysis correlation between selected features. Due to the large number of feature sets, we exclude variables whose correlation coefficient with the outcome variable (*'CBS’*) is less than 0.025 from this figure. There are 26 variables in the figure, meaning that the correlation with the outcome variable is smaller than 0.025 for most variables.

**B. Shapley values of all features**

Figure B.1: SHAP summary plot of all features

**Heterogeneity by country**

Figure B.2: SHAP summary plots

|    1. France |
| --- |
|    1. Italy |
|    1. Spain |
|    1. Germany |
|    1. Sweden |

Figure B.3: Global impacts of the top 30 features in each country

|    1. France |
| --- |
|    1. Italy |
|    1. Spain |
|    1. Germany |
|    1. Sweden |

**C. Robustness check: Feature importance**

Figure C.1: Permutation feature importance of the top 30 feature

**D. Prediction Performance of RF vs OLS**

Table D.1: Accuracy of RF (with 500 trees) with model specific hyperparameters configuration compared with baseline OLS model

| *Prediction performance (MAE and RMSE) on training and testing sample* | | | | | | | | | | |
| --- | --- | --- | --- | --- | --- | --- | --- | --- | --- | --- |
| Model | Metrics | Sample |  | Pooled and per country datasets | | | | | |  |
|  |  |  | Pooled | France | Germany | | Italy | | Spain | Sweden |
| RF | MAE | Trainset  Testset | 0.454  0.461 | 0.473  0.505 | 0.469  0.608 | | 0.412  0.494 | | 0.468  0.581 | 0.468  0.567 |
|  | RMSE | Trainset  Testset | 0.588  0.584 | 0.617  0.652 | 0.603  0.785 | | 0.538  0.621 | | 0.614  0.774 | 0.603  0.720 |
| OLS | MAE | Trainset  Testset | 0.547  0.562 | 0.487  0.542 | 0.571  0.634 | | 0.475  0.497 | | 0.538  0.604 | 0.534  0.594 |
|  | RMSE | Trainset  Testset | 0.711  0.709 | 0.627  0.687 | 0.728  0.834 | | 0.618  0.642 | | 0.696  0.794 | 0.697  0.775 |
|  | *Relative improvement over ordinary least squares* | | | | | | | | |  |
|  | Trainset  MAE Testset | | -17.00%  -17.97% | -2.90%  -6.83% | -17.86%  -4.10% | -13.26%  -0.60% | | -13.01%  -3.81% | | -12.36%  -4.55% |
|  | Trainset  RMSE Testset | | -17.30%  -17.63% | -1.60%  -5.09% | -17.17%  -5.88% | -12.94%  -3.27% | | -11.78%  -2.52% | | -13.49%  -7.10% |

*Notes:* The first part of this table presents the prediction performance of the optimized RF and the baseline OLS model on the pooled and per country samples. The target variable is COVID-19 Behavioral Scale (*CBS*). We optimize the RF using the maximum depth of the trees and the number of features sampled to grow a tree. These hyperparameters have been obtained with a 5-fold cross-validated grid search. The second part of the table shows the relative gain of RF over OLS to reduce prediction errors (measured in MAE and RMSE, see Equation 4 for the definitions).

**E More on Method**

Figure E.1: Overview of overall workflow
